# Comparative Evaluation of Autologous Sticky Bone, Platelet-Rich Fibrin, and Octacalcium Phosphate–Coated Deproteinized Bovine Bone Material for the Regeneration of Human Periodontal Infrabony Defects: Protocol for a Randomized Controlled Clinical Trial

**DOI:** 10.2196/69666

**Published:** 2025-10-10

**Authors:** Sakshi Vishal Kotecha, Priyanka Jaiswal, Shweta Bhagat

**Affiliations:** 1 Department of Periodontics Sharad Pawar Dental College Datta Meghe Institute of Higher Education and Research Wardha India

**Keywords:** autologous sticky bone, ASB, autologous platelet-rich fibrin, autologous PRF, octacalcium phosphate–coated deproteinized bovine bone material, OCP-DBBM, infrabony defects

## Abstract

**Background:**

The term *periodontium* encompasses the supporting structures around a tooth, including gingival tissue, alveolar bone, cementum, and periodontal ligament. Periodontal disorders are highly prevalent, affecting approximately 95% of the Indian population. Restoring the missing attachment apparatus is the main goal of regenerative therapy in cases of periodontal disease. The reproduction or rebuilding of damaged or lost periodontal tissue to restore the structure and functionality of the periodontium is known as regeneration. This study investigates the effectiveness of autologous sticky bone (ASB), autologous platelet-rich fibrin (PRF), and octacalcium phosphate–coated deproteinized bovine bone material (OCP-DBBM). These materials are being evaluated for their potential to enhance bone regeneration in infrabony defects, which are a significant concern in periodontal therapy. Effective bone regeneration is critical for the successful treatment of periodontal defects, as it can lead to improved clinical outcomes, including better attachment levels and reduced probing depths. This study aims to provide insights into the most effective methods for achieving these goals.

**Objective:**

We aim to assess the efficacy of ASB and autologous PRF in conjunction with OCP-DBBM in infrabony defects at 6 months after surgery with regard to radiographic bone fill, reduction in probing pocket depth, and increase in clinical attachment level.

**Methods:**

This randomized controlled clinical trial will be performed on 20 defects in patients diagnosed with stage 2 and 3 grade B periodontitis. It will be a parallel-designed study where group 1 (n=10) will be treated with ASB and group 2 (n=10) will be treated with autologous PRF and OCP-DBBM. One sitting will be required to perform the treatment, and a follow-up checkup will be done at 6 months.

**Results:**

Recruitment procedures started in June 2025. All data are anticipated to be collected by February 2026. Full trial results are anticipated to be analyzed and submitted for publication by March 2026. The study’s anticipated end date is March 2026.

**Conclusions:**

Both treatment approaches are expected to lead to notable gains in periodontal health and bone regeneration. The primary outcome, radiographic bone fill, and the secondary outcomes, clinical attachment level gain and probing pocket depth reduction, are critical indicators of treatment success.

**Trial Registration:**

Clinical Trials Registry-India CTRI/2024/06/069603; https://www.ctri.nic.in/Clinicaltrials/pmaindet2.php?EncHid=MTAzODk0&Enc=&userName=

**International Registered Report Identifier (IRRID):**

PRR1-10.2196/69666

## Introduction

### Background

Disease problems affecting the periodontium are referred to by the word *periodontium*, which describes the supporting structure surrounding a tooth and is made up of the gingival tissue, alveolar bone, cementum, and periodontal ligament. The damaging, irreversible, chronic inflammatory disease that follows gingivitis is called periodontitis. As a result, the bacteria are more likely to enter the surrounding tissues and periodontium. This initiates the host defense system against the microbes that are invading the area [1].

Periodontal disorders, which impact 95% of the Indian population, are one of the most common oral ailments. Periodontitis frequently results in infrabony defects with periodontal pockets, which are the anatomical consequences of plaque spreading apically in the course of periodontitis. “Regeneration of periodontal attachment will become the major goal of regenerative therapy when periodontal disease results in the loss of the attachment apparatus” [2].

Regeneration has been defined as “the reproduction or reconstitution of a lost or injured part to restore the architecture and function of the periodontium. The aim of regenerative periodontal therapy is to restore the structure and function of the periodontium” [3].

Numerous regenerative periodontal therapies have been developed over the past 20 years with the goal of replacing lost tooth-supporting tissues, such as the cementum, alveolar bone, and periodontal ligament [4]. The various regenerative materials used are guided tissue regeneration, enamel matrix derivative, bone grafts like autografts, allografts, and xenografts, matrix-based scaffolds**,** and synthetic materials like demineralized freeze-dried bone allograft (DFDBA), anorganic bovine bone, hydroxyapatite, and tricalcium phosphate. These are osteoconductive in the repair of deformities, acting as a mechanical scaffold for the formation of new bone [5].

When it comes to periodontal graft materials, autograft is regarded as the best. However, patients often resort to alternative periodontal treatment approaches to avoid a surgical donor site and associated complications [6]. Hydroxyapatite, octacalcium phosphate, and DFDBA are a few of the several synthetic materials, xenografts, and allografts that are available and widely used as regeneration materials [7].

Both osteoconductive and osteoinductive qualities are present in the new gold standard for xenografts, macropore octacalcium phosphate–coated deproteinized bovine bone.

This is made from a 100% bovine cancellous bone substitute. Because graft bone materials have a high osteoconductivity, they aid in the proliferation of osteogenic cells and microvessels, which in turn stimulate the formation of new bone [8].

To facilitate the successful regeneration of damaged periodontal tissues, autologous sticky bone (ASB) was introduced as an alternative. ASB is defined as a physiologically solidified bone graft that is embedded in the fibrin network. Strong interconnection of particle bone powders by the fibrin network prevents scattering, even when shaken with a cotton plier. Sticky bone offers many benefits: (1) it can be shaped to suit virtually any kind of bony defects; (2) it prevents the grafted bone from moving; (3) no biochemical additives are needed to maintain the volume of augmentation during the healing period, thereby minimizing the need for block bone and titanium mesh; (4) fibrin accelerates bone and soft tissue regeneration by entrapping platelets and leukocytes that release growth factors; and (5) fibrin interconnection minimizes soft tissue ingrowth into the sticky bone graft [9].

One of the newest developments in regenerative surgery is the use of platelet concentrates for in vivo tissue engineering applications: the first two are platelet-rich fibrin (PRF) and platelet-rich plasma. Platelet concentrates, a concentrated solution of growth factors produced from platelets, are applied as a bioactive surgical adjuvant to facilitate the healing of wounds [10].

As radiographs can identify the quantity and type of damage to the alveolar bone, they play a significant role in periodontal diagnostics. Further research comparing radiographs to presurgical measures found that “bone loss can be underestimated by 1.5 mm, with considerable differences in examiners’ interpretations” [11].

Computed tomography scans have been explored for their potential in highly accurate 3D image alignment. However, they have limitations, including significant radiation exposure and the bulkiness and expense of the equipment [12]. Cone-beam computed tomography (CBCT) has recently emerged as a viable technique in dentistry, offering a lower-cost alternative to traditional computed tomography while still producing high-quality pictures and exposing patients to less radiation.

Therefore, this study will be carried out to evaluate the effectiveness of ASB and autologous PRF along with octacalcium phosphate–coated deproteinized bovine bone material (OCP-DBBM) in the treatment of human infrabony defects.

### Rationale

Periodontitis can lead to the development of infrabony defects and periodontal pockets, resulting in the loss of the attachment apparatus. This can pose a significant problem.

Therefore, the main objective of regenerative therapy is to regenerate the periodontal attachment.

Two distinct approaches will be used: ASB and autologous PRF combined with OCP-DBBM. ASB is a bone graft that hardens physiologically and becomes integrated within the fibrin network. Autologous PRF with OCP-DBBM uses macroporous octacalcium phosphate–coated deproteinized bovine bone. This composite material offers both osteoconductive and osteoinductive properties, making it highly suitable for bone regeneration.

### Objectives

The following are the objectives of this study:

- Objective 1: to assess the efficacy of ASB in infrabony defects at 6 months after surgery with regard to radiographic bone fill, reduction in probing pocket depth (PPD), and increase in clinical attachment level (CAL).
- Objective 2: to assess the efficacy of autologous PRF in conjunction with OCP-DBBM in infrabony defects at 6 months after surgery with regard to radiographic bone fill, reduction in PPD, and increase in CAL.
- Objective 3: to assess the efficacy of ASB and autologous PRF in conjunction with OCP-DBBM in infrabony defects at 6 months after surgery with regard to radiographic bone fill, reduction in PPD, and increase in CAL.

## Methods

### Recruitment

The selection of study participants was performed using well-defined inclusion and exclusion criteria to ensure uniformity and reliability of clinical outcomes. Only systemically healthy individuals diagnosed with chronic periodontitis and presenting with specific radiographic and clinical characteristics were enrolled. The inclusion criteria were designed to identify patients with distinct infrabony periodontal defects that could be assessed radiographically and managed surgically, thereby enabling accurate evaluation of regenerative outcomes. Each participant was selected after completion of initial nonsurgical periodontal therapy and demonstration of acceptable oral hygiene maintenance.

The inclusion parameters emphasized the presence of at least 1 or 2 well-defined infrabony defects with probing depth and clinical attachment loss of ≥5 mm, ensuring adequate disease severity for evaluating regenerative potential. Radiographic criteria, such as a defect depth ≥3 mm and a base positioned ≥3 mm coronal to the root apex, were essential to avoid endodontic involvement and to ensure sufficient bone support for defect containment. The requirement of a minimum of 3 mm of keratinized gingiva around the test tooth ensured soft-tissue stability and favorable surgical access.

The exclusion criteria were framed to eliminate potential confounding variables that could affect periodontal healing or regenerative outcomes. Patients with stage 4 grade C periodontitis, poor plaque control (plaque index >1), or smoking habits were excluded due to their known adverse effects on periodontal regeneration. Similarly, teeth exhibiting mobility greater than grade 2, advanced furcation involvement (class 3 or 4), or prior periodontal surgery in the same quadrant were omitted to maintain site comparability. Pregnant and lactating women were also excluded to prevent any ethical or physiological risks associated with surgical intervention and medication use.

These stringent criteria collectively will ensure that all participants meet the clinical and radiographic prerequisites for reliable assessment of treatment efficacy while minimizing biological and procedural variability (Textbox 1).

The sample size calculation was as follows:

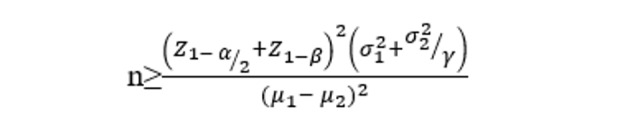



where α=.05, β=.1, SD in group 1 (σ1) is 0.83, mean in group 1 (μ_1_) is 0.522, SD in group 2 is (σ2) 0.044, mean in group 2 (μ_2_) is 0.42, and ratio (group 2 to group 1)=1. The total sample size required was 10 participants per group, as indicated by the calculation [13].

**Inclusion and exclusion criteria for patient screening for infrabony defects.**

**Inclusion criteria**

- Minimally 1 or 2 radiographically identifiable infrabony defects, with a clinical attachment loss and probing pocket depth of ≥5 mm after initial therapy
- Depth of intraosseous component of the defect ≥3 mm
- The radiological base should be ≥3 mm coronal to the tooth’s apex
- The test teeth must have at least 3 mm of keratinized gingiva

**Exclusion criteria**

- Presence of stage 4 grade C periodontitis
- Patients with poor oral hygiene (plaque index>1)
- Habit of smoking
- Study tooth with mobility exceeding grade 2 and exhibiting a class 3 or class 4 furcation defect
- History of periodontal surgical therapy of the selected quadrant
- Women who are pregnant or lactating

Note: The inclusion and exclusion criteria are based on the American Academy of Periodontology 2017 classification [1].

### Intervention Description

Before the surgical procedure, patients will be instructed to rinse their mouths for 1 minute with 0.2% chlorhexidine gluconate solution. Only the oral cavity will be visible because of the patient’s drapes. The patient will remain aseptic for the duration of the process. Using a local anesthetic solution of 2% lidocaine containing 1:100,000 epinephrine (Ligno-Ad local anesthetic, Proxim Remedies), the surgical site will be anesthetized by a nerve block or infiltration anesthesia.

#### Flap Design (Incisions)

A conventional approach consisting of a periodontal access flap will be initiated by intracrevicular (sulcular) incisions using Bard-Parker number 12 or 15 surgical blades on the buccal and lingual aspects. The incisions will be carried as far interproximally as possible to preserve the entire interdental papillae to achieve primary wound closure. A tooth mesial and distal to the defect-associated tooth will be included in the flap. If necessary, divergent vertical relieving incisions will be made one tooth away from the defect, where further access will be required.

#### Flap Reflection

A full-thickness mucoperiosteal flap will be reflected using a periosteal elevator (24G Hu-Friedy) to expose the alveolar bone in the area of the osseous defect. Extreme care will be taken to avoid flap perforation or loss of papillae during removal of granulomatous tissue from the inner aspect of the flap.

#### Debridement and Root Surface Management

The osseous defect will be debrided off the granulation tissue using hand instruments (Gracey curettes, Hu-Friedy) followed by ultrasonic instruments (EMS, Minipiezon), thus exposing the root surface, alveolar bone, and periodontal ligament. Any granulomatous tissue or epithelium adherent to the inner surface of the flap or papillae will be judiciously removed, taking care not to overthin the flap. The root surfaces will be planned to remove plaque and calculus by using ultrasonic instruments, followed by hand instrumentation. The root surfaces will be planed until a smooth and hard consistency is obtained.

At this stage, direct measurements of the vertical bone defects (BDs) and the number of bony walls present will be recorded with the UNC-15 probe. If the BD depth is ≥3 mm vertically, final participant eligibility will be confirmed.

#### Preparation of Autologous PRF

As part of the protocol, approximately 6 mL to 10 mL of venous blood will be collected from the patient’s forearm using an 18G needle in a sterile plastic vacutainer for the preparation of autologous PRF by venipuncture of the median cubital vein and transferred to fresh sterile test tubes and without adding any anticoagulant, the original amount of blood will be divided equally in 2 tubes, which will be placed symmetrically around the rotor axis for proper balancing and centrifuged at 3000 rpm for 10 minutes to produce autologous PRF.

#### Preparation of ASB

Following complete debridement, an 18G needle will be used to draw about 30 mL of venous blood from the patient’s forearm using 1 of 2 vacutainer types: (1) noncoated for autologous fibrin glue and (2) glass coated for concentrated growth factor (CGF) and alternated controlled centrifugation at 2400 rpm to 2700 rpm will be performed for 12 minutes. The autologous fibrin glue obtained will then be mixed with OCP-DBBM for polymerization, which produces a yellow, sticky bone. CGF will be mixed with the prepared sticky bone to produce the CGF–sticky bone mixture.

#### Procedure for Group 1

Complete isolation and hemostasis of the defect will be achieved. The flap will be presutured without tying the knot to allow rapid flap closure after placement of graft material. ASB will be placed into the osseous defect at the test site with light pressure until it is filled to the most coronal level of the osseous wall by raising the flap. The flap will then be coronally repositioned and sutured in such a way that the flap margin will be located 1 mm to 2 mm coronal to the cemento-enamel junction (CEJ), ensuring primary flap closure. A combination of vertical mattress and interproximal sutures (4-0 nonresorbable surgical sutures, braided black silk [Mersilk, Ethicon; Johnson & Johnson Ltd]) will be used to secure the flap in position. Slight pressure will be applied to the area with saline-soaked gauze for approximately 2 minutes to adapt the soft tissue well to the tooth surface and eliminate any space in which a clot might form and disrupt reattachment. The surgical site will be dressed with a periodontal dressing (Coe-Pak, GC Inc) on the buccal and lingual aspects.

#### Procedure for Group 2

The surgical procedure for the defect site will be identical to the procedure of the test site, except that osseous defects at group 2 sites will be packed with OCP-DBBM, and then a PRF membrane will be placed over the bone graft.

#### Maintenance Care

Patients will be recalled after 10 days for suture removal and subsequently for evaluation of both the plaque index (PI) and papillary bleeding index at 3 and 6 months postoperatively. All participants will be reinforced with oral care protocol and full-mouth ultrasonic supragingival scaling during the follow-up visits.

### Statistical Analysis

#### Power

PI, papillary bleeding index, PPD, relative attachment level, and relative gingival marginal level will be calculated by mean and SD values. Statistical tests such as the paired 1-tailed *t* test or Wilcoxon signed-rank test for within-group comparison and the ANOVA or Kruskal-Wallis test for between-group comparison are typically used to assess clinical and radiographic outcomes. The choice of test depends on whether the data follow a normal distribution. The difference between radiographic bone fill, PPD, CAL and relative gingival margin level will be considered significant only if *P*<.05.

The study population will be divided into 2 groups: group 1 (ASB) and group 2 (autologous PRF with OCP-DBBM). There are no subgroups in the study.

#### Evaluation Outcomes

Clinical measurements to be recorded on the day of surgery and 6 months after surgery are PI [14], papillary bleeding index [15], PPD, relative attachment level, and relative gingival marginal level. Periodontal charting on a specially designed form and CBCT will also be documented.

### PI Assessment

Plaque will be assessed on the labial or buccal and lingual surfaces of all the teeth after using a disclosing agent (Table 1) [14].

**Table 1:** Score criteria for the plaque index.

Score	Criteria
0	No plaque
1	Separate flecks of plaque at the cervical margin of the tooth
2	A thin, continuous band of plaque (up to 1 mm) at the cervical margin
3	A band of plaque wider than 1 mm but covering less than one-third of the crown
4	Plaque covering at least one-third but less than two-thirds of the crown
5	Plaque covering two-thirds or more of the crown

### Calculations of PI

The sum of the scores around each tooth was divided by 2 to obtain the PI score for the tooth. The PI score per person will be obtained by totaling all the plaque scores and dividing by the number of teeth examined.

### Papillary Bleeding Index

A periodontal probe will be carefully inserted into the gingival sulcus at the base of the papilla on the mesial aspect and then moved coronally to the papilla tip [15]. This will be repeated on the distal aspect of the same papilla. The intensity of any bleeding thus provoked will be recorded on a 0 to 4 scale (Table 2).

**Table 2:** Score criteria papillary bleeding index.

Score	Criteria
0	No bleeding
1	A single discrete bleeding point appears
2	Several isolated bleeding points or a single fine line of blood appears
3	The interdental triangle fills with blood shortly after probing
4	Profuse bleeding occurs after probing; blood flows immediately into marginal sulcus

### Calculations of Papillary Bleeding Index

Each papilla will be scored according to the aforementioned criteria. The sum of all scores will be divided by the number of papillae examined to obtain the papillary bleeding index score per person.

### Probing Measurements

Both treatment groups will be subjected to recordings to measure PPD, relative CAL, and relative gingival margin level at 6 sites of the selected teeth. Measurements will be carried out using a UNC-15 calibrated periodontal probe (University of North Carolina) at the mesiobuccal, mesiolingual, distobuccal, distolingual, midbuccal, and midlingual points of the selected tooth. After measurement, only the deepest marking per defect will be considered for study purposes. After the fabrication of the acrylic stent, probing measurements will be recorded at baseline and 6 months postoperatively.

The UNC-15 probe will be positioned on the prepared groove of the acrylic stent. The probe will be placed with its tip at the gingival marginal level, and the distance between the gingival margin and the lower border of the stent will be measured as the relative gingival margin level. Then, the probe will be positioned at the bottom of the pocket, and the distance from the lower border of the stent to the bottom of the pocket will be noted as the relative CAL. PPD will be measured by subtracting the relative gingival margin level from the relative CAL. The width of keratinized gingiva will be calculated from the lowest point of the mucogingival junction to the crest of the gingival margin using the UNC-15 probe.

### Radiographic Analysis

The infrabony defect sites will be investigated with CBCT at baseline and 6 months postoperatively. The CBCT analysis will include the measurement of BD height (CEJ–base of the defect), level of alveolar crest (AC; CEJ–AC), BD depth (AC-BD), and the mesiodistal (MD) and buccolingual BD width. The landmark of the base of the defect will be the lowest discontinuous point of the periodontal ligament. A line perpendicular from the AC to the root surface will be drawn, and the intersection point across the root surface will be termed as AC'.

Infrabony defect depth: The distance from the point AC' to the base of the defect (AC'-BD). MD width of the infrabony defect (AC-AC): The distance from the point AC' to the AC. The buccolingual width of the defect will be measured by selecting the innermost and most coronal points of the buccal and lingual AC on the axial plane, and calculating the horizontal distance between these two points. A 1.00-mm incremental slice thickness will be used in this study’s investigation, as smaller slices decrease the image resolution. In the oblique view, the x-, y-, and z-axes will be sequentially analyzed to locate the most apical point of the BD or the most coronal aspect of the defect-associated AC [6].

### Plans to Promote Participant Retention and Complete Follow-Up

Patients will be recalled after surgery at 1, 3, and 6 months. At every recall appointment, all participants will receive guidance on maintaining good oral hygiene as well as full-mouth professional prophylaxis, which involves ultrasonic scaling.

### Ethical Considerations

This study received approval on January 30, 2024, from the Institutional Ethics Committee of Datta Meghe Institute of Higher Education and Research (DMIHER DU/IEC/2024/227) to ensure adherence to ethical guidelines, respect participant rights, and meet established standards. This study has been registered with the Clinical Trials Registry-India under registration CTRI/2024/06/069603. Participants will be provided with an informed consent form, clearly describing the study’s aim, procedures, risks, benefits, and their right to opt out at any time. Consent for publication of data will be obtained and will be used for all analyses, including secondary analyses. We will confirm that the original consent and institutional review board approval explicitly cover the use of primary and secondary data. Collection of data and entering the data into the database for screening and randomization will be done by the primary investigator. The privacy of prospective and enrolled participants’ personal information will be upheld to ensure confidentiality before, during, and after the trial.We ensure that the data collected will be fully anonymized, with no personally identifiable information retained or linked to any participant. As there are no interventions involved and as only a cross-sectional survey will be conducted in which the data will be collected via an interview and anthropometric measurements, participants will not be provided with any compensation in this study.

## Results

It is anticipated that ASB will show better results. The study aims to evaluate the effectiveness of different materials in the regeneration of human periodontal infrabony defects. The principal outcomes are presented subsequently.

### Primary Outcome: Radiographic Bone Fill

The primary outcome measured in the study is radiographic bone fill at 6 months after surgery. This outcome is crucial as it indicates the extent of bone regeneration achieved through the different treatment modalities being tested.

### Secondary Outcomes

In addition to the primary outcome, the study also assesses secondary outcomes, which include the following:

- CAL gain, which measures the improvement in the attachment of the periodontal tissue to the tooth, indicating successful regeneration.
- PPD reduction, which is used to evaluate the decrease in the depth of periodontal pockets, which is a sign of improved periodontal health.
- Relative gingival marginal level, which has been documented to assess the health of the gingival tissue surrounding the teeth.

## Discussion

### Anticipated Findings

It is anticipated that the use of ASB will show a better outcome in treating infrabony defects because of its advantageous properties provided by the strong fibrin interconnection with particulate bone powder.

ASB, a composite graft derived from a mixture of bone particles and platelet-rich plasma, has been previously studied for its osteoinductive and osteoconductive properties, contributing to enhanced periodontal regeneration. This study aligns with previous reports demonstrating the ability of ASB to promote significant periodontal tissue regeneration and improved clinical outcomes, particularly in terms of bone fill and attachment gain [16]. The osteoconductive nature of ASB, combined with its growth factor–enriched environment, likely facilitates the recruitment of stem cells and fibroblasts, fostering tissue remodeling and bone regeneration [17].

PRF, obtained through centrifugation of autologous blood to isolate a fibrin matrix rich in platelets, has also been shown to enhance healing in periodontal defects through the release of growth factors and cytokines that promote cellular proliferation and angiogenesis [18]. Similar findings have been reported by Jee [19], who showed that PRF accelerates the healing process in periodontal tissue by facilitating the regeneration of the bone matrix and soft tissue. While both ASB and PRF have exhibited favorable outcomes, the superior regenerative capacity of ASB in terms of bone fill and attachment gain may be attributed to the additional presence of bone graft material, which enhances the scaffold effect and provides more robust osteoconductive support [20].

OCP-DBBM is a biomaterial composed of deproteinized bovine bone mineral coated with octacalcium phosphate, which has demonstrated promising potential for bone regeneration in periodontal defects because of its osteoconductive properties [21]. While OCP-DBBM contributed to moderate bone fill and clinical attachment gain, the lack of autologous growth factors may limit its regenerative potential compared with PRF and ASB, which both leverage endogenous healing factors.

It is also worth considering that the clinical success of biomaterials such as OCP-DBBM is often contingent on the patient’s biological response, which may vary between individuals. The relatively lower efficacy of OCP-DBBM in comparison to the other two regenerative modalities may be attributed to the absence of a direct cellular component, limiting the healing potential to the osteoconductive properties of the material alone.

### Mechanisms of Regeneration

The regenerative success of both ASB and PRF can be attributed to the synergistic effects of their cellular and biochemical components. By combining platelet-rich plasma with autologous bone graft particles, sticky bone creates a scaffold that promotes osteogenic differentiation and tissue integration by releasing growth factors like platelet-derived growth factor, vascular endothelial growth factor, and transforming growth factor-β [22]. These growth factors play a critical role in enhancing osteoblastic differentiation and angiogenesis, key processes necessary for effective bone regeneration.

Similarly, PRF’s effectiveness stems from its natural fibrin matrix, which facilitates the slow release of growth factors that contribute to soft tissue healing, as well as bone formation [19]. The combination of PRF with bone grafts has been found to produce a superior regenerative outcome compared with bone grafts alone, likely because of the enhanced cellular activity and tissue remodeling it promotes [23].

In contrast, OCP-DBBM primarily provides a physical scaffold that supports bone growth through osteoconductive mechanisms, relying on the native healing process of the host tissue. Although OCP-DBBM has shown efficacy in bone regeneration, it may not provide the same level of biological support as ASB and PRF, which actively participate in healing through growth-factor processes and cell-mediated processes.

### Clinical Implications

The findings of this study underscore the importance of selecting appropriate regenerative materials based on defect characteristics and patient needs. For patients with complex periodontal defects, the combination of autologous biological materials like ASB and PRF may offer a more favorable outcome because of the incorporation of growth factors and cellular components that promote tissue regeneration. These treatments may be particularly beneficial in cases where rapid healing and significant bone regeneration are paramount.

However, for less complex defects or in cases where the use of autologous materials is not feasible, OCP-DBBM provides a viable alternative, offering satisfactory regeneration with a lower risk of complications and easier handling.

### Limitations

This study has several potential limitations:

- A small sample size may not accurately reflect the larger population and may restrict how broadly the findings can be applied.
- Longer follow-up periods could provide more comprehensive insights into the long-term effectiveness and stability of the treatments used.
- The results may not apply to different kinds of bone graft materials or periodontal regeneration methods.
- No histomorphometric analysis will be performed.

### Comparison With Prior Work

Singh and Kashyap [13] conducted a clinical study comparing ASB and a mixture of autologous PRF and bioactive glass (PRF-BG) for regenerating periodontal infrabony defects. They found that, unlike other platelet concentrates, PRF and CGF do not need external anticoagulants and release growth factors that aid healing. However, CGF and PRF alone cannot retain bone graft material long enough for proper healing. To improve bone graft stability, they tested 3 therapies (open flap debridement [OFD], PRF-BG, and ASB). The graft materials were well tolerated, and PRF-BG and ASB groups showed reduced postoperative inflammation compared with the OFD group.

Najeeb et al [8] conducted a clinical study comparing the resolution of infrabony defects and bone regeneration in three-walled defects treated with OFD using either OCP-DBBM (Ti-oss) or DFDBA. Based on CBCT analysis, they concluded that Ti-oss demonstrated superior regenerative outcomes.

### Conclusions

The outcomes of this investigation may yield critical insights into the comparative efficacy of therapeutic modalities for periodontal regeneration, particularly in the management of infrabony defects. Such evidence could inform and refine clinical decision-making, contributing to enhanced patient-centered outcomes, including reductions in probing depth, gains in CAL, radiographic evidence of osseous fill, and the establishment of more efficacious and standardized treatment protocols within the field of periodontics.

### Future Research Directions

The study may also highlight the need for further research to explore long-term outcomes and the effectiveness of these treatments in diverse patient populations. This could help with refining techniques and improving overall treatment strategies in periodontal therapy.

## Abbreviations

AC: alveolar crest
ASB: autologous sticky bone
BD: bone defect
CAL: clinical attachment level
CEJ: cemento-enamel junction
CBCT: cone-beam computed tomography
CGF: concentrated growth factor
DFDBA: demineralized freeze-dried bone allograft
MD: mesiodistal
OCP-DBBM: octacalcium phosphate–coated deproteinized bovine bone material
OFD: open flap debridement
PI: plaque index
PPD: probing pocket depth
PRF: platelet-rich fibrin
PRF-BG: platelet-rich fibrin and bioactive glass

## Data Availability

Data sharing is not applicable to this article as no data sets were generated or analyzed during this study.
